# Neurotransmitter CART as a New Therapeutic Candidate for Parkinson’s Disease

**DOI:** 10.3390/ph6010108

**Published:** 2013-01-18

**Authors:** Peizhong Mao, Charles K. Meshul, Philippe Thuillier, P. Hemachandra Reddy

**Affiliations:** 1 Department of Physiology and Pharmacology, Oregon Health and Science University, Portland, OR 97239, USA; 2 Public Health and Preventive Medicine, and the Knight Cancer Institute, Oregon Health and Science University, Portland, OR 97239, USA; 3 Division of Neuroscience, Oregon National Primate Research Center, Oregon Health and Science University, Beaverton, OR 97006, USA; 4 Research Services, Portland VA Medical Center, Portland, OR 97239, USA; 5 Departments of Behavioral Neuroscience and Pathology, Oregon Health and Science University, Portland, OR 97239, USA

**Keywords:** cocaine- and amphetamine- regulated transcript, mitochondria, antioxidant, dopamine, oxidative stress, neuroprotection

## Abstract

Parkinson’s disease (PD) is one of the most common neurodegenerative diseases. To date, there is no effective treatment that halts its progression. Increasing evidence indicates that mitochondria play an important role in the development of PD. Hence mitochondria-targeted approaches or agents may have therapeutic promise for treatment of the disease. Neuropeptide CART (cocaine-amphetamine-regulated transcript), a hypothalamus and midbrain enriched neurotransmitter with an antioxidant property, can be found in mitochondria, which is the main source of reactive oxygen species. Systemic administration of CART has been found to ameliorate dopaminergic neuronal loss and improve motor functions in a mouse model of PD. In this article, we summarize recent progress in studies investigating the relationship between CART, dopamine, and the pathophysiology of PD, with a focus on mitochondria-related topics.

## 1. Introduction

Parkinson’s disease (PD), the second most common neurodegenerative disorder after Alzheimer’s disease (AD), is a severe, progressive disease that affects approximately 1-2% of persons older than 40 years of age [1]. Clinically, it is characterized by muscle rigidity, tremor, a slowing of physical movement, and in extreme cases, a loss of physical movement. In addition, PD is commonly viewed as an extrapyramidal motor disorder that results primarily from the death of dopaminergic neurons in the substantia nigra (SN) of the ventral midbrain [2,3,4,5,6,7].

The etiology of PD is complicated and ultimately remains unknown. However, mitochondrial dysfunction is known to play a key role in the development of PD, with PD recognized as one of the putative mitochondrial diseases [7]. Interestingly, sporadic and familial PD seem to converge at the level of mitochondrial integrity [8,9]. Since mitochondria are the major source of reactive oxygen species (ROS), and have a crucial role in cellular bioenergetics and apoptosis, as well as PD pathogenesis, mitochondria-related therapeutics may open new avenues for the treatment of the disease.

Recently, we found that the brain-rich neuropeptide CART (cocaine- and amphetamine- regulated transcript) is preferentially localized to mitochondria with both mitochondria activating and antioxidative properties *in vitro and in vivo* [10,11]. These findings suggest that CART may be a new candidate for therapeutic agents targeting PD. In this article, we summarize recent progress made in investigating characteristics of CART as a potential therapeutic agent for PD, focusing on CART in relationship to mitochondria function.

## 2. Etiology and Pathophysiology of PD

Although the etiology of PD is unclear, both genetic and environmental factors appear to play a role [4,12,13]. Genetic changes are involved in the development of PD, but only 0.5-10% of PD cases can be attributed to mutations in specific genes. The remaining 90-99.5% of idiopathic PD cases are sporadic [2,8,14].

### 2.1. Genetic Factors

Several genes and several putative loci have been identified as causal factors of PD [14,15,16,17,18]. Two autosomal-dominant mutations of the α-synuclein (αSyn) gene (also known as SNCA and PARK1) were discovered and linked to the rare familial, early-onset PD [19]. αSyn was subsequently shown to be the major component of Lewy bodies (LBs), a hallmark feature of PD [20,21]. Even sporadic PD cases have also been genetically linked to αSyn polymorphisms, which affect αSyn expression at the transcription level [22]. The extent to which nigral and cortical LBs are present in the PD brain is uncertain. Although cortical LBs may be detected in most PD patients, it is unknown how many are necessary to cause dopamine neuron death, as well as cognitive changes. It is possible that both the distribution (cortex versus midbrain/brainstem) and the density of these lesions interact to produce specific clinical phenotypes. It has now been widely accepted that accumulations of the pathologic species of αSyn and the deposition of αSyn fibrils and ubiquitinated proteins into inclusions of αSyn, at least partially result in motor, cognitive and behavioral phenotypes [23,24]. The current evidence indicates that the inhibition of mitochondrial complex I, *in vitro* and *in vivo*, may lead to accumulation of αSyn inclusions, suggesting that αSyn aggregation is a downstream consequence of mitochondrial dysfunction and might be an effecter of neuronal cell death [26,27,28].

Interestingly, both Parkin (PARK2) and PARK5 are ubiquitin–proteasome related proteins that are involved in the proteasomal degradation of αSyn [15,25]. Mutations in PARK2 are associated with PD [29]. PARK5 encodes UCHL1 (ubiquitin carboxyl-terminal hydrolase isozyme L1), a deubiquitinating enzyme that is responsible for making ubiquitin. Ubiquitin is required for the ubiquitin-proteasome pathway in neurons to target proteins for degradation, and the point mutation of PARK5 is associated with PD [15,30]. However, the association between UCHL1/PARK5 and PD is still controversial.

Mutations in the putative mitochondrial proteins PINK1 (PTEN-induced kinase 1, PARK6) and DJ-1 (PARK7) have been linked to familial forms of PD [31,32,33]. These wild-type genes have a mitochondria protective role against oxidative stress [34,35].

Mutations in the LRRK2 (PARK8) gene are, to date, the most common in both familial and sporadic PD [18]. Recently, it was found that the G2019S mutation can cause uncoupling protein-mediated mitochondrial depolarization via a cell-specific increase in uncoupling protein (UCP) 2 and 4 expression [36].

Therefore several causal genes for PD have been reported and they will be further investigated for confirmation of their role in the disease and the related molecular mechanisms. In addition, more PD-associated genes may be identified via powerful methods such as genome-wide association studies (GWAS) in the future. Interestingly, many of these PD-related genes are ubiquitously expressed in the brain where they have been reported to have many important functions. Speculations have been offered to explain how mutations in such genes lead to a loss of dopaminergic neurons in the midbrain [2,3,5,25,37]. One proposal suggests that mitochondria are exposed to oxidative stress (see 2.2 for details) as a by-product of dopamine metabolism. Another proposed idea suggests a problem in maintaining mitochondria and cellular function in the elongated processes of these neurons, including intracellular calcium overload.

Interestingly, many motor symptoms of PD result from the death of substantia nigra pars compacta (SNc) dopaminergic neurons. However, there is a host of other symptoms that have been linked to PD pathology. This includes significant cognitive changes and dementia in PD patients, the latter may be at least partially due to memory-related neuronal loss in the hippocampus.

### 2.2. Mitochondrial Dysfunction and Oxidative Stress

Accumulating data indicate that mitochondrial dysfunction, particularly complex I inhibition and oxidative stress play a major role in the pathogenesis of PD [16,25,38,39,40,41]. Since the early 1980s, inhibition of complex of oxidative phosphorylation (OXPHOS) has been found to induce pathologic symptoms of PD, mitochondrial dysfunction, and oxidative stress [42,43]. Decrements in complex I activity in the substantia nigra of sporadic PD patients [38,44] and some chemicals, such as MPTP (1-methyl-4-phenyl-1,2,3,6-tetrahydropyridine), 6-hydroxydopamine (6-OHDA) and rotenone, have also been found to inhibit the electron transport in OXPHOS and to replicate most features of PD in humans and in animal models [45,46,47,48,49,50].

Notably, results from recent work suggest that several PD-associated genes interface with pathways regulating mitochondrial function, morphology, and dynamics [7,9,16,51]. For example, an intriguing relationship between mitochondria and αSyn indicated that αSyn aggregation is a downstream consequence of mitochondrial dysfunction [26,28,52].

Ca^2+^ homeostasis is also regulated by mitochondria. Elevated cytosolic Ca^2+^ stimulates mitochondrial respiratory metabolism and ROS generation, and eventually induces dopaminergic cell death. Studies demonstrating that the Ca^2+^ channel blocker protects dopamine neurons *in vitro* and *in vivo* further indicate the importance of Ca^2+^ homeostasis in the pathology of PD [53,54]. Depletion of glutathione levels (the most abundant antioxidant in the human body) is the earliest biochemical event to occur in the Parkinsonian substantia nigra prior to selective loss of complex I activity. Recent studies have demonstrated that reduction in both cellular and mitochondrial glutathione levels results in increased oxidative stress and a decrease in mitochondrial function. This has been linked to a selective decrease in complex I activity, suggesting the potential therapeutic role of antioxidants for treatment of PD [55,56].

The substantia nigra is a midbrain center composed of dopamine and other neurons. The dopamine system plays a key role in the control or regulation of locomotion, learning, working memory, cognition, and emotion [57,58]. Mitochondrial monoamine oxidase (MAO, especially MAO-B in humans) is responsible for the breakdown of dopamine. Its enzymatic activity (monoamine degradation) induces a number of neurotoxic species, particularly hydrogen peroxide, which forms highly reactive hydroxyl radicals, leading to oxidative stress, additional mitochondria damage, and neuronal degeneration [59,60,61].

Post-mortem brain studies have consistently implicated oxidative damage in the pathogenesis of PD. In particular, oxidative damage to lipids, proteins, and DNA has been observed in the substantia nigra of sporadic PD brains [62,63,64,65]. The source of this increased oxidative stress is unclear but may include mitochondrial dysfunction, increased dopamine metabolism that can yield excess hydrogen peroxide and other ROS as described above, an increase in reactive iron, and impaired antioxidant defense pathways [3,39].

Overall, there is extensive evidence suggesting a major role for mitochondrial dysfunction in the pathogenesis of PD and, in particular, defects in mitochondrial complex I of the respiratory chain. A complex I defect could most obviously contribute to neuronal degeneration in PD through decreased ATP synthesis and could cause damage by excess ROS production. Hence, the improvement of mitochondrial function could be a basic and important strategy to delay or to prevent neuronal cell death in PD patients.

## 3. CART Is a New Peptide Hormone with Multiple Functions

In 1995, using a polymerase chain reaction (PCR) differential display, researchers discovered the neuropeptide CART while searching for mRNAs in the striatum that had been acutely up-regulated by psychostimulants [66]. In rat, the primary CART transcript is differentially spliced, and the two different mRNAs encode peptides of either 116 or 129 amino acids. The leader sequence consists of 27 amino acids, and the mature CART peptides therefore contain either 89 or 102 residues. CART peptide processing is tissue-dependent and two forms predominate the rat brain: CART (42–89 a.a.) and CART (49–89 a.a.) [67]. CART is also expressed in all levels of the hypothalamic-pituitary-adrenal (HPA) axis [68,69], which plays an important role in energy homeostasis and the neuroendocrine response to stress [70,71]. CART is a unique and an important peptide hormone that affects multiple physiological and pathological processes, such as stress response, food intake and body weight control, and regulation of neuroendocrine functions [11,72,73,74]. CART is also related to human health problems, including anxiety and depression, drug abuse, obesity and diabetes [71,74,75,76]. In addition, CART exhibited a transcriptional function when the conserved C-terminus of CART was fused to the GAL4 DNA-binding domain [77]. Gene expression profiling with gene microarrays revealed that CART mRNA was strongly enhanced by estradiol in an ischemic animal model. In addition, the CART peptide exhibited neuroprotective properties against ischemic brain injury *in vivo* and against oxygen–glucose-deprivation (OGD)-induced cell death in primary cortical neurons. This neuroprotective effect of CART has been linked to ERK activation and to the upregulation of brain-derived neurotrophic factor (BDNF) [78,79,80]. Finally, CART may be a useful biomarker for some human diseases, such as dementia with LBs, which is believed to be a syndrome in both Alzheimer’s disease and PD [81,82].

## 4. New Functions of CART Related to Mitochondria

Despite the many known functions of CART peptides, its mechanisms of action remain a mystery. A major issue is that no interaction partners or receptors have been reported. To address this problem and determine the mechanism(s) of CART’s actions, we used a yeast two-hybrid system and found the first partner for CART: succinate dehydrogenase (SDH, also known as respiratory chain complex II). The interaction was also confirmed by a pull down assay [10]. SDH is a functional member of both the Krebs cycle and the aerobic respiratory chain (complex I-V) within the mitochondria and is crucial to normal intermediary metabolism and energy production in most cells. Complex II couples the oxidation of succinate to fumarate in the mitochondrial matrix with the reduction of ubiquinone in the membrane [83]. Complex II is composed of two hydrophilic subunits, a flavoprotein (SDHA) , an iron-sulfur protein (SDHB) subunit, and two hydrophobic membrane anchor subunits, SDHC and SDHD [83,84]. Mutations in SDHB and depression of SDH activity have been strongly implicated in neuronal aging [85] and in development of CNS tumors [86,87].

### 4.1. CART Stimulates the Activities of SDH and Complex II, Increases Cellular ATP Levels

Our studies suggest that CART stimulates the activities of SDH and complex II under basal and OGD conditions, and reduces neuronal cell death *in vitro* and *in vivo*. Given the important role that mitochondria play in neuronal function, survival, and protection of CART from ischemia, our results strongly suggest an interaction between CART and SDH as a mechanism of neuroprotection [10].

**Figure 1:**
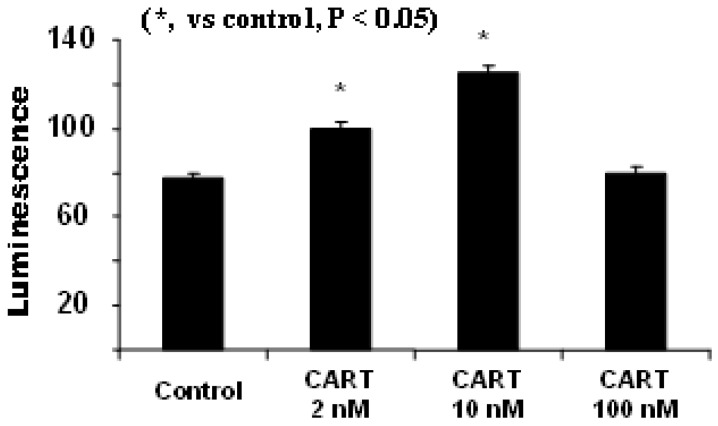
CART increases ATP synthesis in purified neuronal mitochondria. Mitochondria were extracted from cultured primary cortex neuronal cells and treated directly with CART peptide or vehicle (control). ATP levels were measured using a luciferase-based bioluminescence detection assay.

Because CART increases the activity of the SDH/complex II, we hypothesized that CART affects the production of ATP, the final step of energy production in the organelles. To investigate the direct effects of CART on mitochondrial ATP levels, we isolated mitochondria from the primary cortical neurons, and then added CART or a vehicle to the mitochondria. We found that 2 nM and 10 nM of CART significantly stimulated ATP production in the isolated mitochondria (Figure 1), but high concentrations of CART (e.g., 100 nM) had no stimulatory effect. However, maximum ATP production was achieved with 10 nM CART treatment. Subsequently, we tested the protective effects of CART in a pathological/hypoxic condition. Using primary neuronal culture (*in vitro* days 10-12), we added CART or a vehicle to the media; 30-min later we replaced the media with an oxygen-depleted, glucose- and serum-free buffer, and then incubated the cultures for 2 hours in an anoxia chamber filled with an anoxic gas mixture containing 5% H_2_, 5% CO_2_, and 90% N_2_ at 37°C. Immediately after OGD, we replaced the media with fresh feeding media containing glucose and CART or the vehicle, and returned the cells to normoxia for 24 hours. We isolated cell mitochondria and measured ATP levels. The results showed that OGD treatment caused a remarkable reduction in ATP levels, while CART treatments inhibited reduction of ATP levels. We also observed a dose-dependent increase in ATP production [10]. These effects are consistent with molecular genetics results in which CART was found to target the cellular mitochondrial respiratory chain complex II (SDH). A similar result was also observed in neuronal cells that were treated with a mitochondrial toxin and in human embryonic kidney cells treated with hydrogen peroxide [10,11]. Therefore, CART may be a mitochondrial activator in mammalian and human cells, and it may have a clinical application for the treatment of some human diseases, such as PD.

### 4.2. CART’s Antioxidant Properties

**Figure 2:**
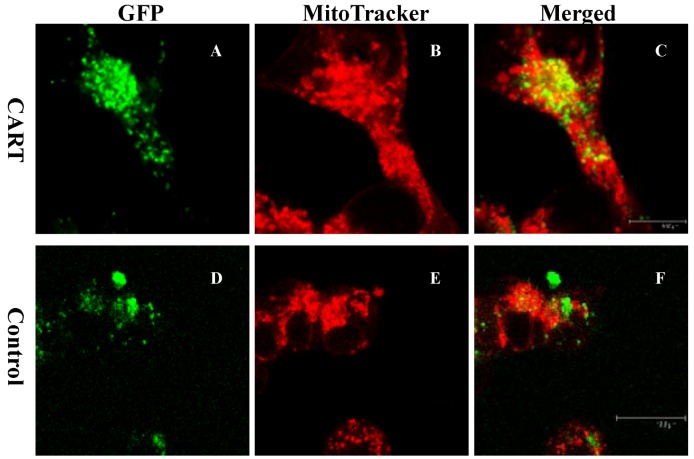
TATEGFP-CART mainly localized in the mitochondria. Cultured HEK293 cells were treated overnight with TATEGFP-CART or TATEGFP (control) fusion proteins, and then treated with the mitochondria marker MitoTracker Red for 30 min. Living cells were analyzed by a confocal microscopy. A-C, confocal fluorescence images depict cells treated with TATEGFP-CART fusion proteins; and D-F, cells treated with vehicle TATEGFP fusion proteins (control). Pixels containing fluorescence for both GFP (green) and the red (mitochondria) appear as yellow in the merged images, indicating co-localization. TATEGFP-CART (C) was preferentially localized into mitochondria compared to TATEGFP (F) [11].

Recent research revealed that CART has several antioxidant properties [11]. Oxidative damage that had been induced by H_2_O_2_ was significantly reduced in the presence of CART. In particular, mtDNA damage, lipid peroxidation, oxidized proteins, and cell death were significantly inhibited when CART was expressed; this response was dose-dependent. Interestingly, as shown in Figure 2, localization of CART was tracked to the mitochondria in HEK 293 cells, as well as in cultured primary cortex neurons and hippocampal neurons [11]. Studies in the mouse brain further confirmed that CART was localized in the inner and outer membranes of mitochondria. The localization of CART to the mitochondria and its strong antioxidant activity further support the interaction of CART with SDH [10]. Direct antioxidant and radical-scavenging activities of CART were further demonstrated by its ability to inhibit peroxyl radicals that induced the oxidation of cis-parinaric acid. As little as 1 nM of CART was efficacious in inhibiting this oxidation, and 20 nM CART almost completely prevented peroxidation. CART’s antioxidant activities and subcellular localization may have clinical significance, especially in the context of neuron degenerative disorders in which mitochondrial dysfunction may be implicated.

### 4.3. CART Is Physiologically Associated with the Dopamine System

Dopaminergic neurons of the midbrain are the main source of dopamine in the mammalian central nervous system. Although their numbers are few, dopaminergic neurons play an important role in the control of multiple brain functions, including voluntary movement and a broad array of behavioral processes, such as mood, reward, addiction, and stress [88]. These functions are also regulated by hypothalamus hormones/peptides including CART.

Studies into developmental pathways that are involved in the generation of dopaminergic neurons in the brain have identified a connection between dopaminergic neurons and CART. CART was originally identified as an mRNA that increases in the rat striatum after acute cocaine or amphetamine administration. The highest CART expression levels in murine, monkey, and human brains were detected in the hypothalamus. In addition, CART transcript is also predominantly expressed in target regions of the mesocorticolimbic dopamine system [71,89].

The distribution and synaptic connectivity of CART peptides in the ventral midbrain were investigated via histochemistry analyses. Immunoreactive (CARTir) terminal varicosities were located throughout the rostrocaudal extent of the substantia nigra, the ventral tegmental, and retrorubral field. Very interestingly, they were particularly abundant in the dorsomedial substantia nigra where they overlapped with proximal dendrites of dopaminergic SNc neurons. CARTir terminals were also found in dopaminergic perikarya in the ventromedial part of the rostral SNc [90]. CART projections, were mostly localized in the ventral midbrain and synaptic interactions with dopamine neurons [91].

A close interaction between CART and dopamine systems has also been observed in the nucleus accumbens. The release of CART from the axonal terminals in this region may serve as the final output of the endogenous opioid-mesolimbic-dopamine circuitry that processes natural reward [92]. In addition, CART affects locomotor activity through simultaneous stimulation of D1 and D2 dopamine receptors [93]. Interestingly, the release of CART peptides was found to increase coordinately following increased dopaminergic activity in animals exposed to a novel, conspecific social environment [94].

To understand the link between CART and dopaminergic neurons, a more detailed immunohistochemistry of the rat mesencephalon was performed and compared to the expression of tyrosine hydroxylase, a marker for dopamine. CART neurons were first detected on embryonic day 11 in the ventral mesencephalic vesicle [95]. Many tyrosine hydroxylase-positive neurons were found to migrate from the neuroepithelium through the area containing the CARTir neurons and settled more laterally. These tyrosine hydroxylase cells exhibited prominent leading and trailing dendrites in the immediate vicinity of CART perikarya. Thus, these data suggest that CART-producing neurons might help the migration of the substantia nigra dopaminergic neurons [95], and indicate interaction between endogenous CART and dopamine neurons.

### 4.4. The Role of CART in PD Model

Using a neuroprotective mouse model for PD, we found that pre-treatment of mice with CART, followed by daily administration of the neurotoxin MPTP for 7 days, resulted in a reduced loss of dopamine nerve cells in the substantia nigra pars compacta, compared to mice that were treated only with the MPTP [11]. This reduced loss of dopamine neurons was associated with improvement in behavior in both rearing and reduced footfaults. In the future, to determine whether CART will either slow down or reverse the effects of MPTP, CART will need to be administered either during or after the last dose of MPTP. Subsequently, a recently developed progressive model of dopamine cell and terminal loss [96], will need to be tested. For further investigation of CART actions and specificity, a mutant CART peptide or antisense CART peptide will need to be tested as a scramble control at the same time. Morphologically, CART was located within nerve terminals making a symmetrical synaptic contact, consistent with the hypothesis that this peptide may be associated with dopamine-containing nerve terminals. In addition, in only those terminals or dendrites that were positive for CART immuno-labeling, the outer membrane of mitochondria was also labeled. It is therefore possible that the neuroprotective effects of CART could be due to CART protecting mitochondria against MPTP toxicity by supplying sufficient ATP levels at nerve terminals.

### 4.5. CART as a Potential Therapeutic Target for PD

**Figure 3:**
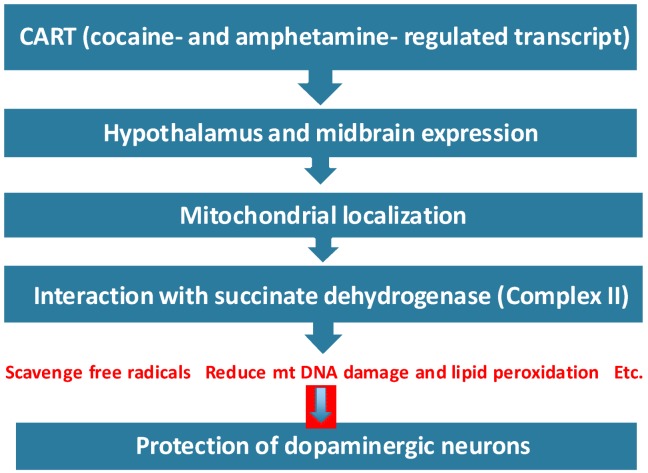
Possible roles for CART in Parkinson’s disease. CART peptide is widely expressed in the brain, especially in the hypothalamus and midbrain. CART is localized in mitochondria, interacts with SDH, and increases cellular ATP. CART also scavenges excessive free radicals that are predominantly generated in dopamine neurons via mitochondrial MAO-B in PD. Consequently, CART could be involved in the reduction of oxidative damages of mtDNA, lipids and proteins, and CART could eventually protect dopamine neurons from degradation.

As discussed above, CART is a multi-functional hormone, mainly expressed in the brain, particularly in the dopamine system-associated hypothalamus and midbrain. Based on the localization of CART in cells and its expression levels there, CART hormone may play an important role in several physiological and pathological processes, including endocrine regulation, body weight control, and the onset of anxiety and depression [71,74]. Our recent data demonstrated that, in the cellular matrix, CART acts as a strong antioxidant that targets mitochondria. Interestingly, CART crosses the blood-brain barrier easily [11]. More importantly, CART was also found in the outer membrane of mouse brain neurons, where MAO enzymes are localized [11,61]. We recently found that CART affects several mammalian and human cells: 1) CART interacts with the complex II protein, succinate dehydrogenase SDH, and this interaction is reported to enhance mitochondrial protection via ATP biogenesis; 2) it protects mtDNA and cellular lipids and proteins against oxidative insults, 3) it scavenges mitochondrial ROS, 4) it protects dopamine neurons in a mouse model of PD [10,11]. These findings suggest that CART may function via mechanisms as shown in Figure 3, in which case CART may be a promising drug to target dopaminergic neurons in PD patients.

Currently, there are no drugs or agents that delay and/or prevent disease progression in PD patients (and in the elderly population). As discussed earlier, mitochondrial (particularly complex I) dysfunction and electrons leakage/oxidative stress play a large role in PD pathogenesis. Thus, accepting or feeding electrons into mitochondrial electron transport chain through complex II may be a beneficial approach in patients with Parkinson’s disease. The discovery of energy-biogenesis for CART peptide and its antioxidant protective properties against dopamine neurons suggests a possible therapeutic application for PD as well as other mitochondrial dysfunction-related diseases. However, further research is still needed to understand the precise mechanisms by which CART protects mitochondria and its role in mitochondrial biogenesis, mitochondrial axonal transport and synaptic protection (particularly in dopamine neurons), and its relationship with PD causative genes. In terms of the involvement of CART in therapeutic strategies for PD patients, endogenous mitochondrial CART levels could be increased in PD patients through endocrine mechanisms and/or other approaches such as mitochondria-targeted CART overexpression in dopamine neurons. These strategies may protect dopamine neurons from oxidative and toxic insults from mutant protein(s), resulting in the delay of disease progression in PD patients.

## 5. Conclusions

The cause of PD, one of the most common neurodegenerative diseases, is unknown, and there is no cure or effective treatment. Mitochondria, the powerhouse of the cell, have diverse functions and properties and could be critically involved in the development of PD. Structural and biochemical data from studies of central nervous systems from post-mortem human brains, as well as cell and animal models of PD, suggest that mitochondrial dysfunction is a trigger or propagator of neurodegeneration in PD.

To date, several key genes associated with PD have been identified, and many of these genes are ubiquitously expressed with important functions in the brain. The genes related to PD, and their products play a role in mitochondrial functions, suggesting that these gene products share a common pathway at the mitochondrial level toward nigral degeneration in both familial and sporadic PD.

Mitochondrial dysfunction and oxidative stress are the by-products of the dopamine metabolism that leads to the death of dopamine neurons and may be involved in the etiology of PD. Hence, there is increasing interest in the administration of antioxidants that can target mitochondria and ROS scavengers in treatment of this progressive neurodegenerative disease. CART is believed to protect mitochondria by the interaction with the key mitochondrial enzyme SDH and may be involved in mitochondria-related diseases, especially PD. Recent studies of CART that have revealed CART’s mitochondrial protection warrant further investigation of CART as a potential treatment for PD and perhaps other degenerative diseases.

## References

[1] Tanner C.M., Goldman S.M. (1996). Epidemiology of Parkinson's Disease. Neurol. Clin..

[2] Barzilai A., Melamed E. (2003). Molecular Mechanisms of Selective Dopaminergic Neuronal Death in Parkinson's Disease. Trends Mol. Med..

[3] Moore D.J., West A.B., Dawson V.L., Dawson T.M. (2005). Molecular Pathophysiology of Parkinson's Disease. Annu. Rev. Neurosci..

[4] Thomas B., Beal M.F. (2007). Parkinson's Disease. Hum. Mol. Genet..

[5] Banerjee R., Starkov A.A., Beal M.F., Thomas B. (2009). Mitochondrial Dysfunction in the Limelight of Parkinson's Disease Pathogenesis. Biochim. Biophys. Acta.

[6] Schapira A.H. (2012). Targeting Mitochondria for Neuroprotection in Parkinson's Disease. Antioxid. Redox Signal..

[7] Schapira A.H. (2012). Mitochondrial Diseases. Lancet.

[8] Wood-Kaczmar A., Gandhi S., Wood N.W. (2006). Understanding the Molecular Causes of Parkinson's Disease. Trends Mol. Med..

[9] Winklhofer K.F., Haass C. (2010). Mitochondrial Dysfunction in Parkinson's Disease. Biochim. Biophys. Acta.

[10] Mao P., Ardeshiri A., Jacks R., Yang S., Hurn P.D., Alkayed N.J. (2007). Mitochondrial Mechanism of Neuroprotection by CART. Eur. J. Neurosci..

[11] Mao P., Meshul C.K., Thuillier P., Goldberg N.R., Reddy P.H. (2012). CART Peptide is a Potential Endogenous Antioxidant and Preferentially Localized in Mitochondria. PLoS One.

[12] Warner T.T., Schapira A.H. (2003). Genetic and Environmental Factors in the Cause of Parkinson's Disease. Ann. Neurol..

[13] Schapira A.H. (2009). Etiology and Pathogenesis of Parkinson Disease. Neurol. Clin..

[14] Schapira A.H. (2006). Etiology of Parkinson's Disease. Neurology.

[15] Lansbury P.T., Brice A. (2002). Genetics of Parkinson's Disease and Biochemical Studies of Implicated Gene Products. Curr. Opin. Cell Biol..

[16] Dodson M.W., Guo M. (2007). Pink1, Parkin, DJ-1 and Mitochondrial Dysfunction in Parkinson's Diseas. Curr. Opin. Neurobiol..

[17] Nuytemans K., Theuns J., Cruts M., Van Broeckhoven C. (2010). Genetic Etiology of Parkinson Disease Associated with Mutations in the SNCA, PARK2, PINK1, PARK7, and LRRK2 Genes: A Mutation Update. Hum. Mutat..

[18] Martin L.J. (2012). Biology of Mitochondria in Neurodegenerative Diseases. Prog. Mol. Biol. Transl. Sci..

[19] Polymeropoulos M.H., Lavedan C., Leroy E., Ide S.E., Dehejia A., Dutra A., Pike B., Root H., Rubenstein J., Boyer R. (1997). Mutation in the Alpha-Synuclein Gene Identified in Families with Parkinson's Disease. Science.

[20] Spillantini M.G., Schmidt M.L., Lee V.M., Trojanowski J.Q., Jakes R., Goedert M. (1997). Alpha-Synuclein in Lewy Bodies. Nature.

[21] Uversky V.N., Li J., Bower K., Fink A.L. (2002). Synergistic Effects of Pesticides and Metals on the Fibrillation of Alpha-Synuclein: Implications for Parkinson's Disease. Neurotoxicology.

[22] Maraganore D.M., de Andrade M., Elbaz A., Farrer M.J., Ioannidis J.P., Kruger R., Rocca W.A., Schneider N.K., Lesnick T.G., Lincoln S.J. (2006). Collaborative Analysis of Alpha-Synuclein Gene Promoter Variability and Parkinson Disease. JAMA.

[23] Galvin J.E., Pollack J., Morris J.C. (2006). Clinical Phenotype of Parkinson Disease Dementia. Neurology.

[24] Galvin J.E. (2006). Cognitive Change in Parkinson Disease. Alzheimer Dis. Assoc. Disord..

[25] Janda E., Isidoro C., Carresi C., Mollace V. Defective Autophagy in Parkinson's Disease: Role of Oxidative Stress. Mol. Neurobiol..

[26] Betarbet R., Sherer T.B., MacKenzie G., Garcia-Osuna M., Panov A.V., Greenamyre J.T. (2000). Chronic Systemic Pesticide Exposure Reproduces Features of Parkinson's Disease. Nat. Neurosci..

[27] Langston J.W., Sastry S., Chan P., Forno L.S., Bolin L.M., Di Monte D.A. (1998). Novel Alpha-Synuclein-Immunoreactive Proteins in Brain Samples from the Contursi Kindred, Parkinson's, and Alzheimer's Disease. Exp. Neurol..

[28] Forno L.S. (1996). Neuropathology of Parkinson's Disease. J. Neuropathol. Exp. Neurol..

[29] Kitada T., Asakawa S., Hattori N., Matsumine H., Yamamura Y., Minoshima S., Yokochi M., Mizuno Y., Shimizu N. (1998). Mutations in the Parkin Gene Cause Autosomal Recessive Juvenile Parkinsonism. Nature.

[30] Giasson B.I., Lee V.M. (2003). Are Ubiquitination Pathways Central to Parkinson's Disease?. Cell.

[31] Valente E.M., Abou-Sleiman P.M., Caputo V., Muqit M.M., Harvey K., Gispert S., Ali Z., Del Turco D., Bentivoglio A.R., Healy D.G. (2004). Hereditary Early-Onset Parkinson's Disease Caused by Mutations in PINK1. Science.

[32] Silvestri L., Caputo V., Bellacchio E., Atorino L., Dallapiccola B., Valente E.M., Casari G. (2005). Mitochondrial Import and Enzymatic Activity of PINK1 Mutants Associated to Recessive Parkinsonism. Hum. Mol. Genet..

[33] Bonifati V., Rizzu P., van Baren M.J., Schaap O., Breedveld G.J., Krieger E., Dekker M.C., Squitieri F., Ibanez P., Joosse M. (2003). Mutations in the DJ-1 Gene Associated with Autosomal Recessive Early-Onset Parkinsonism. Science.

[34] Matsuda N., Sato S., Shiba K., Okatsu K., Saisho K., Gautier C.A., Sou Y.S., Saiki S., Kawajiri S., Sato F. (2010). PINK1 Stabilized by Mitochondrial Depolarization Recruits Parkin to Damaged Mitochondria and Activates Latent Parkin for Mitophagy. J. Cell Biol..

[35] Guzman J.N., Sanchez-Padilla J., Wokosin D., Kondapalli J., Ilijic E., Schumacker P.T., Surmeier D.J. (2010). Oxidant Stress Evoked by Pacemaking in Dopaminergic Neurons is Attenuated by DJ-1. Nature.

[36] Papkovskaia T.D., Chau K.Y., Inesta-Vaquera F., Papkovsky D.B., Healy D.G., Nishio K., Staddon J., Duchen M.R., Hardy J., Schapira A.H. (2012). G2019S Leucine-Rich Repeat Kinase 2 Causes Uncoupling Protein-Mediated Mitochondrial Depolarization. Hum. Mol. Genet..

[37] Abeliovich A. (2010). Parkinson's Disease: Mitochondrial Damage Control. Nature.

[38] Schapira A.H., Mann V.M., Cooper J.M., Dexter D., Daniel S.E., Jenner P., Clark J.B., Marsden C.D. (1990). Anatomic and Disease Specificity of NADH CoQ1 Reductase (Complex I) Deficiency in Parkinson's Disease. J. Neurochem..

[39] Jenner P. (2003). Oxidative Stress in Parkinson's Disease. Ann. Neurol..

[40] Lin M.T., Beal M.F. (2006). Mitochondrial Dysfunction and Oxidative Stress in Neurodegenerative Diseases. Nature.

[41] Moran M., Moreno-Lastres D., Marin-Buera L., Arenas J., Martin M.A., Ugalde C. (2012). Mitochondrial Respiratory Chain Dysfunction: Implications in Neurodegeneration. Free Radic. Biol. Med..

[42] Jenner P. (1993). Presymptomatic Detection of Parkinson's Disease. J. Neural Transm. Suppl..

[43] Zhou C., Huang Y., Przedborski S. (2008). Oxidative Stress in Parkinson's Disease: A Mechanism of Pathogenic and Therapeutic Significance. Ann. N. Y. Acad. Sci..

[44] Schapira A.H., Cooper J.M., Dexter D., Clark J.B., Jenner P., Marsden C.D. (1990). Mitochondrial Complex I Deficiency in Parkinson's Disease. J. Neurochem..

[45] Dauer W., Przedborski S. (2003). Parkinson's Disease: Mechanisms and Models. Neuron.

[46] Gunzler S.A., Shakil S., Carlson N.E., Nutt J.G., Meshul C.K. (2007). Low Doses of Apomorphine Transiently Reduce Locomotor Activity in MPTP-Treated Mice. Neurosci. Lett..

[47] Meshul C.K., Emre N., Nakamura C.M., Allen C., Donohue M.K., Buckman J.F. (1999). Time-Dependent Changes in Striatal Glutamate Synapses Following a 6-Hydroxydopamine Lesion. Neuroscience.

[48] Robinson S., Freeman P., Moore C., Touchon J.C., Krentz L., Meshul C.K. (2003). Acute and Subchronic MPTP Administration Differentially Affects Striatal Glutamate Synaptic Function. Exp. Neurol..

[49] Fox S.H., Brotchie J.M. (2010). The MPTP-Lesioned Non-Human Primate Models of Parkinson's Disease. Past, Present, and Future. Prog. Brain Res..

[50] Iderberg H., Francardo V., Pioli E.Y. (2012). Animal Models of L-DOPA-Induced Dyskinesia: An Update on the Current Options. Neuroscience.

[51] Wilhelmus M.M., Nijland P.G., Drukarch B., de Vries H.E., van Horssen J. (2012). Involvement and Interplay of Parkin, PINK1, and DJ1 in Neurodegenerative and Neuroinflammatory Disorders. Free Radic. Biol. Med..

[52] Manning-Bog A.B., McCormack A.L., Li J., Uversky V.N., Fink A.L., Di Monte D.A. (2002). The Herbicide Paraquat Causes Up-Regulation and Aggregation of Alpha-Synuclein in Mice: Paraquat and Alpha-Synuclein. J. Biol. Chem..

[53] Dawson T.M., Dawson V.L. (2003). Molecular Pathways of Neurodegeneration in Parkinson's Disease. Science.

[54] Chan C.S., Guzman J.N., Ilijic E., Mercer J.N., Rick C., Tkatch T., Meredith G.E., Surmeier D.J. (2007). Rejuvenation' Protects Neurons in Mouse Models of Parkinson's Disease. Nature.

[55] Chinta S.J., Andersen J.K. (2006). Reversible Inhibition of Mitochondrial Complex I Activity Following Chronic Dopaminergic Glutathione Depletion in Vitro: Implications for Parkinson's Disease. Free Radic. Biol. Med..

[56] Chinta S.J., Kumar M.J., Hsu M., Rajagopalan S., Kaur D., Rane A., Nicholls D.G., Choi J., Andersen J.K. (2007). Inducible Alterations of Glutathione Levels in Adult Dopaminergic Midbrain Neurons Result in Nigrostriatal Degeneration. J. Neurosci..

[57] Sarkar C., Basu B., Chakroborty D., Dasgupta P.S., Basu S. (2010). The Immunoregulatory Role of Dopamine: An Update. Brain Behav. Immun..

[58] Rasheed N., Alghasham A. (2012). Central Dopaminergic System and its Implications in Stress-Mediated Neurological Disorders and Gastric Ulcers: Short Review. Adv. Pharmacol. Sci..

[59] Hastings T.G. (2009). The Role of Dopamine Oxidation in Mitochondrial Dysfunction: Implications for Parkinson's Disease. J. Bioenerg. Biomembr..

[60] Mao P., Reddy P.H. Aging and Amyloid Beta-Induced Oxidative DNA Damage and Mitochondrial Dysfunction in Alzheimer's Disease: Implications for Early Intervention and Therapeutics. Biochim. Biophys. Acta.

[61] Duncan J., Johnson S., Ou X.M. (2012). Monoamine Oxidases in Major Depressive Disorder and Alcoholism. Drug Discov. Ther..

[62] Alam Z.I., Jenner A., Daniel S.E., Lees A.J., Cairns N., Marsden C.D., Jenner P., Halliwell B. (1997). Oxidative DNA Damage in the Parkinsonian Brain: An Apparent Selective Increase in 8-Hydroxyguanine Levels in Substantia Nigra. J. Neurochem..

[63] Alam Z.I., Daniel S.E., Lees A.J., Marsden D.C., Jenner P., Halliwell B. (1997). A Generalised Increase in Protein Carbonyls in the Brain in Parkinson's but Not Incidental Lewy Body Disease. J. Neurochem..

[64] Jenner P. (2007). Oxidative Stress and Parkinson's Disease. Handb. Clin. Neurol..

[65] Seet R.C., Lee C.Y., Lim E.C., Tan J.J., Quek A.M., Chong W.L., Looi W.F., Huang S.H., Wang H., Chan Y.H. (2010). Oxidative Damage in Parkinson Disease: Measurement using Accurate Biomarkers. Free Radic. Biol. Med..

[66] Douglass J., McKinzie A.A., Couceyro P. (1995). PCR Differential Display Identifies a Rat Brain mRNA that is Transcriptionally Regulated by Cocaine and Amphetamine. J. Neurosci..

[67] Thim L., Kristensen P., Nielsen P.F., Wulff B.S., Clausen J.T. (1999). Tissue-Specific Processing of Cocaine- and Amphetamine-Regulated Transcript Peptides in the Rat. Proc. Natl. Acad. Sci. U. S. A..

[68] Larsen P.J., Seier V., Fink-Jensen A., Holst J.J., Warberg J., Vrang N. (2003). Cocaine- and Amphetamine-Regulated Transcript is Present in Hypothalamic Neuroendocrine Neurones and is Released to the Hypothalamic-Pituitary Portal Circuit. J. Neuroendocrinol..

[69] Koylu E.O., Couceyro P.R., Lambert P.D., Ling N.C., DeSouza E.B., Kuhar M.J. (1997). Immunohistochemical Localization of Novel CART Peptides in Rat Hypothalamus, Pituitary and Adrenal Gland. J. Neuroendocrinol..

[70] Koylu E.O., Balkan B., Kuhar M.J., Pogun S. (2006). Cocaine and Amphetamine Regulated Transcript (CART) and the Stress Response. Peptides.

[71] Mao P. (2011). Potential Antidepressant Role of Neurotransmitter CART: Implications for Mental Disorders. Depress Res. Treat..

[72] Kuhar M.J., Adams S., Dominguez G., Jaworski J., Balkan B. (2002). CART Peptides. Neuropeptides.

[73] Hunter R.G., Kuhar M.J. (2003). CART Peptides as Targets for CNS Drug Development. Curr. Drug Targets CNS Neurol. Disord..

[74] Rogge G., Jones D., Hubert G.W., Lin Y., Kuhar M.J. (2008). CART Peptides: Regulators of Body Weight, Reward and Other Functions. Nat. Rev. Neurosci..

[75] del Giudice E.M., Santoro N., Cirillo G., D'Urso L., Di Toro R., Perrone L. (2001). Mutational Screening of the CART Gene in Obese Children: Identifying a Mutation (Leu34Phe) Associated with Reduced Resting Energy Expenditure and Cosegregating with Obesity Phenotype in a Large Family. Diabetes.

[76] Yanik T., Dominguez G., Kuhar M.J., Del Giudice E.M., Loh Y.P. (2006). The Leu34Phe ProCART Mutation Leads to Cocaine- and Amphetamine-Regulated Transcript (CART) Deficiency: A Possible Cause for Obesity in Humans. Endocrinology.

[77] Mao P., Jacks R. (2007). Transcriptional Activity by Cocaine-Amphetamine-Regulated Transcript. Mol. Psychiatry.

[78] Wu B., Hu S., Yang M., Pan H., Zhu S. (2006). CART Peptide Promotes the Survival of Hippocampal Neurons by Upregulating Brain-Derived Neurotrophic Factor. Biochem. Biophys. Res. Commun..

[79] Xu Y., Zhang W., Klaus J., Young J., Koerner I., Sheldahl L.C., Hurn P.D., Martinez-Murillo F., Alkayed N.J. (2006). Role of Cocaine- and Amphetamine-Regulated Transcript in Estradiol-Mediated Neuroprotection. Proc. Natl. Acad. Sci. USA.

[80] Zhang M., Han L., Xu Y. (2012). Roles of Cocaine- and Amphetamine-Regulated Transcript in the Central Nervous System. Clin. Exp. Pharmacol. Physiol..

[81] Mao P. (2012). Recent progress and concerns in dementia: distinguishing Alzheimer's disease and dementia with Lewy bodies via biochemical markers in the cerebrospinal fluid. Advances in Biological Chemistry.

[82] Swerdlow R.H., Newell K.L. (2012). "Untangling" the Relationship between Alzheimer Disease and Dementia with Lewy Bodies. Neurology.

[83] Cecchini G. (2003). Function and Structure of Complex II of the Respiratory Chain. Annu. Rev. Biochem..

[84] Rustin P., Rotig A. (2002). Inborn Errors of Complex II--Unusual Human Mitochondrial Diseases. Biochim. Biophys. Acta.

[85] Bertoni-Freddari C., Fattoretti P., Paoloni R., Caselli U., Galeazzi L., Meier-Ruge W. (1996). Synaptic Structural Dynamics and Aging. Gerontology.

[86] Gimenez-Roqueplo A.P., Favier J., Rustin P., Rieubland C., Kerlan V., Plouin P.F., Rotig A., Jeunemaitre X. (2002). Functional Consequences of a SDHB Gene Mutation in an Apparently Sporadic Pheochromocytoma. J. Clin. Endocrinol. Metab..

[87] Maier-Woelfle M., Brandle M., Komminoth P., Saremaslani P., Schmid S., Locher T., Heitz P.U., Krull I., Galeazzi R.L., Schmid C. (2004). A Novel Succinate Dehydrogenase Subunit B Gene Mutation, H132P, Causes Familial Malignant Sympathetic Extraadrenal Paragangliomas. J. Clin. Endocrinol. Metab..

[88] Chinta S.J., Andersen J.K. (2005). Dopaminergic Neurons. Int. J. Biochem. Cell Biol..

[89] Fagergren P., Hurd Y. (2007). CART mRNA Expression in Rat Monkey and Human Brain: Relevance to Cocaine Abuse. Physiol. Behav..

[90] Dallvechia-Adams S., Smith Y., Kuhar M.J. (2001). CART Peptide-Immunoreactive Projection from the Nucleus Accumbens Targets Substantia Nigra Pars Reticulata Neurons in the Rat. J. Comp. Neurol..

[91] Dallvechia-Adams S., Kuhar M.J., Smith Y. (2002). Cocaine- and Amphetamine-Regulated Transcript Peptide Projections in the Ventral Midbrain: Colocalization with Gamma-Aminobutyric Acid, Melanin-Concentrating Hormone, Dynorphin, and Synaptic Interactions with Dopamine Neurons. J. Comp. Neurol..

[92] Upadhya M.A., Nakhate K.T., Kokare D.M., Singh U., Singru P.S., Subhedar N.K. (2012). CART Peptide in the Nucleus Accumbens Shell Acts Downstream to Dopamine and Mediates the Reward and Reinforcement Actions of Morphine. Neuropharmacology.

[93] Moffett M.C., Song J., Kuhar M.J. (2011). CART Peptide Inhibits Locomotor Activity Induced by Simultaneous Stimulation of D1 and D2 Receptors, but Not by Stimulation of Individual Dopamine Receptors. Synapse.

[94] Hostetler C.M., Kowalczyk A.S., Griffin L.L., Bales K.L. (2011). CART Peptide Following Social Novelty in the Prairie Vole (Microtus Ochrogaster). Brain Res..

[95] Brischoux F., Griffond B., Fellmann D., Risold P.Y. (2002). Early and Transient Ontogenetic Expression of the Cocaine- and Amphetamine-Regulated Transcript Peptide in the Rat Mesencephalon: Correlation with Tyrosine Hydroxylase Expression. J. Neurobiol..

[96] Goldberg N.R., Haack A.K., Lim N.S., Janson O.K., Meshul C.K. (2011). Dopaminergic and Behavioral Correlates of Progressive Lesioning of the Nigrostriatal Pathway with 1-Methyl-4-Phenyl-1,2,3,6-Tetrahydropyridine. Neuroscience.

